# Sustaining dry surfaces under water

**DOI:** 10.1038/srep12311

**Published:** 2015-08-18

**Authors:** Paul R. Jones, Xiuqing Hao, Eduardo R. Cruz-Chu, Konrad Rykaczewski, Krishanu Nandy, Thomas M. Schutzius, Kripa K. Varanasi, Constantine M. Megaridis, Jens H. Walther, Petros Koumoutsakos, Horacio D. Espinosa, Neelesh A. Patankar

**Affiliations:** 1 Department of Mechanical Engineering, Northwestern University, Evanston, IL, USA; 2 Institute of Computational Science, ETH Zürich, Zürich, Switzerland; 3 School for Engineering of Matter, Transport and Energy, Arizona State University, Tempe, AZ, USA; 4 Department of Mechanical and Industrial Engineering, University of Illinois at Chicago, Chicago, IL, USA; 5 Department of Mechanical Engineering, MIT, Cambridge, MA, USA; 6 Department of Mechanical Engineering, Tech. University of Denmark, Kgs. Lyngby, Denmark

## Abstract

Rough surfaces immersed under water remain practically dry if the liquid-solid contact is on roughness peaks, while the roughness valleys are filled with gas. Mechanisms that prevent water from invading the valleys are well studied. However, to remain practically dry under water, additional mechanisms need consideration. This is because trapped gas (e.g. air) in the roughness valleys can dissolve into the water pool, leading to invasion. Additionally, water vapor can also occupy the roughness valleys of immersed surfaces. If water vapor condenses, that too leads to invasion. These effects have not been investigated, and are critically important to maintain surfaces dry under water. In this work, we identify the critical roughness scale, below which it is possible to sustain the vapor phase of water and/or trapped gases in roughness valleys – thus keeping the immersed surface dry. Theoretical predictions are consistent with molecular dynamics simulations and experiments.

Superhydrophobicity occurs when surface roughness enhances non-wetting properties of hydrophobic solids^1^,^2^. Maintaining superhydrophobicity of rough textured surfaces has typically relied on the presence of trapped air pockets in the roughness valleys^3^. Keeping these surfaces practically dry (liquid minimally touching the solid surface) under water is challenging because the trapped air is found to deplete^4^,^5^,^6^,^7^,^8^,^9^,^10^. This depletion limits the utility of these surfaces in applications like drag reduction^4^,^5^,^11^, boiling^12^, among others. We investigate how immersed surfaces can remain practically dry. We postulate that it is essential to stabilize the vapor phase of water and sustain trapped gases in roughness valleys. There is a critical roughness scale, below which these mechanisms are effective. These are passive thermodynamic mechanisms that do not involve active generation^5^ or exchange of gas^13^,^14^. We show that surfaces of hydrophobic solids retain non-wetting properties in the presence of sub-micrometer roughness. Theoretical predictions are consistent with molecular dynamics simulations, experiments, and observations of air-retaining insect surfaces^15^,^16^. It is our intention that this work will pave way to rationally design surface texture to manipulate the phase of one material adjacent to a surface – in this instance acquiring a vapor phase between a liquid and a textured solid surface, even when the liquid is not heated or boiled.

Although dry immersed rough surfaces may be achieved, the underlying mechanisms that drive non-wetting to wetting transitions are not fully understood. Research on the well-known wetting behavior of non-immersed rough surfaces^1^,^2^, manifested in the form of liquid droplets beading up and moving with very little drag, has intensified in recent years^3^. In this case, the droplets reside on top of roughness peaks, while air occupies roughness valleys. This is the Cassie-Baxter state^1^. Maintaining the Cassie-Baxter state will ensure a practically dry surface while immersed in a liquid. This is challenging^4^,^5^,^6^,^7^,^9^, as air in the roughness valleys can dissolve into the liquid if the liquid is undersaturated with air. Thus, in order to keep a surface practically dry under water, the gas phase in the roughness valleys must be sustained.

The thermodynamic analysis of underwater superhydrophobicity that accounts for only the surface energy has been theoretically studied^17^. To make robust surfaces that remain dry under water, the effect of sustaining vapor pockets also needs to be accounted in the thermodynamic analysis^18^,^19^. To elucidate the fundamental principles required to sustain gas pockets, we consider a typical cylindrical pore on a surface that is immersed under water. When the surface is immersed under water, there will initially be air trapped in the pore (roughness valley). For this air to be sustained over a long period, it should be in chemical equilibrium with air dissolved in the ambient liquid. If the liquid is supersaturated with air, an air layer covering the surface may be achieved indefinitely^13^. However, if the liquid is undersaturated, then air within the pore will dissolve into the liquid^20^. Consequently, air pressure inside the pore will decline, and water will invade if the liquid-air interface cannot remain pinned at the top of the pore^21^,^22^,^23^,^24^,^25^,^26^,^27^,^28^,^29^,^30^,^31^,^32^,^33^,^34^,^35^. The invading liquid will lead to the wetting of the immersed surface.

Trapped air is not the only gas that can occupy the pore. At temperatures below the boiling point, the liquid phase is the lower energy state. However, a metastable vapor can evaporate from the meniscus (hanging at the top of the pore) and occupy the pore. This vapor inside the pore could eventually condense on the pore walls, thus providing another pathway, via condensation, to wet the pore. Will the metastable vapor occupy the pore and keep it dry or will it condense in the pore to make it wet? This is a critical consideration, hitherto unresolved, and which is essential to enabling practically dry surfaces immersed in undersaturated liquids.

We term the phenomenon of sustaining the metastable vapor in the pore as vapor-stabilization. This is important because it permits sustaining the vapor phase without actually having to boil the liquid. This mechanism has been considered to stabilize the film-boiling mode even at low superheats^36^. Analysis of the energetics of the competing scenarios (wetting vs. non-wetting) leads to the following condition to avoid liquid invasion and keep the pore dry^18^,^19^:


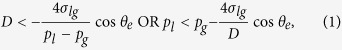


where *D* is the pore diameter, *p*_*l*_ the liquid pressure, *p*_*g*_ the pressure of the gas in the pore, 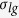 the liquid-gas surface energy, and *θ*_*e*_ the equilibrium contact angle of a liquid drop on a flat solid surface of the same material as the pore. Typically, the gas will be a combination of trapped air and the vapor phase of the liquid, both of which should be in chemical equilibrium with the dissolved air in the liquid and the liquid itself, respectively (see Supplementary Figures S1-S2)^18^,^19^. Here, the pore is assumed to be deep enough so that the curved liquid-gas interface, hanging at the top of the pore, does not touch the bottom^24^. Equation (1) shows that for a given liquid pressure, the pore diameter should be smaller than a critical value to keep the pore dry. It is emphasized that the condition in equation (1) ensures two scenarios: First, that the liquid does not impale from the top of the pore (a well-known result from before^37^), and second, that the vapor itself does not condense inside the pore to fill it up from within. The latter condition can be understood as follows. For condensation to occur within the pore, a drop of condensate of a critical size must form according to heterogeneous nucleation theory^38^,^39^. However, if the pore size is small enough (equation (1)), then the drop of condensate starts wetting the pore walls before it reaches a critical size. Wetting the pore wall is energetically expensive if the wall is hydrophobic. Hence, the energy barrier for condensation increases. This would prevent the vapor from condensing within the pore and filling it up. Thus, equation (1) plays a dual role of preventing impalement, as well as condensation of the liquid inside the pore (see derivation of the condensation-based criterion in Supplementary Section 1). For example, assume that all air has dissolved out of the pore due to undersaturation of the liquid and the only gas in the pore is vapor in chemical equilibrium with liquid water at 300 K and standard atmospheric pressure. This represents a vapor-stabilized scenario that would keep the pore dry. In this case, *p*_*l*_ = 101.325 kPa, *p*_*g*_ ≈ 3.539 kPa^18^,^19^, 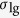 = 71.7 mN/m^40^, and 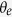 = 110° (typical value attained by hydrophobic chemical coatings) yield a critical pore diameter of 1 μm. Equation (1) can also be used to predict the liquid pressure, above which the vapor will not be stabilized and liquid invasion will occur.

Based on the above analysis, we predict that practically dry rough surfaces are possible in water, even after trapped air has fully depleted, due to the stabilized vapor phase of the liquid in the roughness valley. We estimate that, for typical liquid pressures, this will be feasible for pore diameters (roughness spacing) that are hundreds of nanometers or less, but not for roughness scales of tens of microns or larger. These conclusions based on pore-type geometries can be extended to pillar-type geometries without fundamental difficulty^18^,^19^. In the remaining sections, we verify the above predictions using molecular dynamics simulations, experiments, and observations of air-retaining insects.

## Results

### Simulations

**Figure 1:**
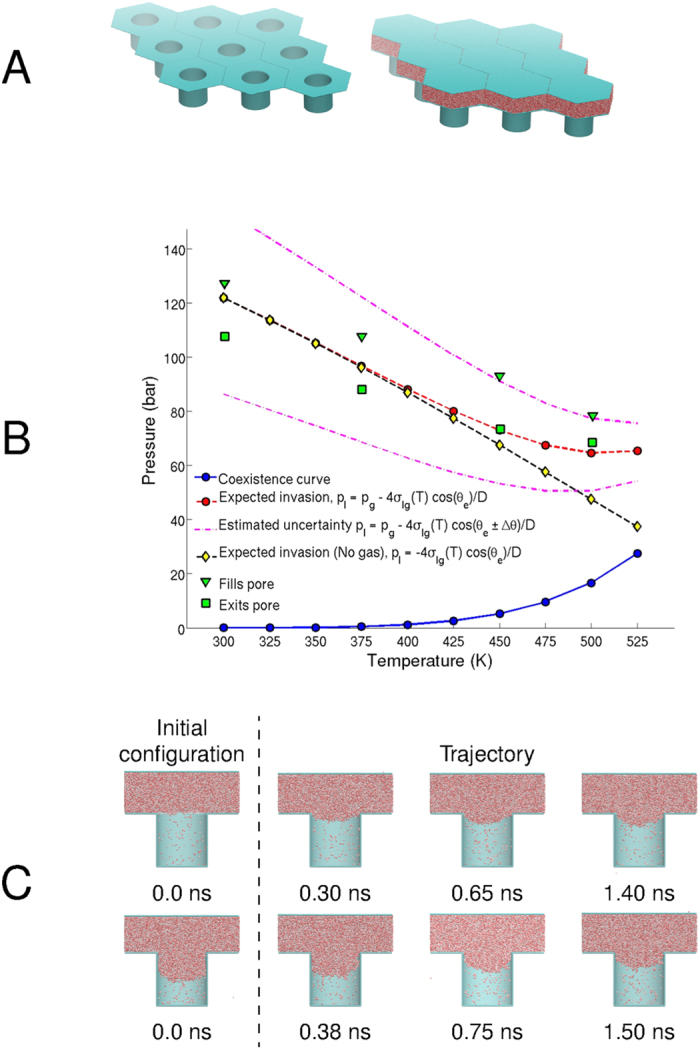
(**A**) Molecular dynamics model of a cylindrical pore surface with periodic boundary conditions. Water is placed on top of the textured surface. A rigid surface (piston) is used to apply pressure to the liquid water. (**B**) Liquid-vapor phase diagram for pore simulations. Stabilization and invasion pressures applied by the piston for an initially unfilled and initially half-filled pore were the same. The coexistence curve of the SPC/E water model obtained from the publicly available NIST Standard Reference Simulation Website^57^ is shown. Expected liquid invasion pressures were determined by equation (1) using a calculated liquid-solid contact angle of θ_e_ = 119.4°, with surface energies obtained from Sakamaki *et al.*^58^ Upper and lower estimates of the liquid invasion pressure were made using equation (1) with contact angle (θ_e_ ± Δθ), where Δθ = 9.07°. (**C**) Molecular dynamics simulations of a hydrophobic pore demonstrating non-wetting at 501 K and 68.59 bar applied pressure. The top row simulation begins with an unfilled pore; the bottom row simulation begins with a half-filled pore. The final state is the same for each case – dry.

Molecular dynamics simulations using NAMD^41^ 2.9 software were used to verify the liquid invasion pressures predicted using equation (1). To simulate an immersed rough surface, a 10 nm diameter cylindrical pore is assembled using VMD^42^ software, with periodic boundary conditions for the overall domain (Fig. 1A). The pore is solvated with SPC/E^43^ water molecules residing initially outside the pore (on top of the roughness peaks). A rigid surface (piston) is used to apply pressure to the liquid water pooled above the pore. The pore assembly and meniscus trajectories are shown in Fig. 1 (also see Supplementary Figures S4-S5). The molecular dynamics results for invasion pressures applied at the piston, compared with theoretical predictions from equation (1), are shown in Fig. 1B for temperatures of 300 K, 375 K, 450 K, and 501 K. For each temperature (from low to high), the corresponding pressures: *p*_*l*_ = 107.79 bar, 88.19 bar, 73.49 bar, and 68.59 bar, respectively, demonstrate a resistance to liquid invasion, and hence, an immersed surface that remains practically dry. At the same respective temperatures, but higher applied pressures, liquid invades the pore. A temperature of 501 K is used to allow a significant amount of vapor to accrue within the pore. A contact angle of 119.4° (accurate to within 9.07°) is determined from the angle between the meniscus and vertical pore walls at a temperature of 300 K (see Supplementary Section 2 for details). To demonstrate robustness against liquid invasion into the pore, we additionally simulate pores that are initially half-filled with water. The non-wetting behavior of these pores is consistent with simulations of water initially outside the pore. This is shown in Fig. 1C for two simulations at a temperature of 501 K and 68.59 bar liquid pressure. We also note that at this temperature and pressure, the liquid is below its boiling point; yet the pore becomes occupied by the metastable vapor, as predicted. This method of using texture to control phase may potentially be extended to other phase transformations of water as well. In fact, molecular dynamics simulations indicate that condensation, or full wetting, can be achieved using rough hydrophilic surfaces at conditions above the boiling point of water (see Supplementary Figure S6). At these conditions, the presence of vapor is expected due to boiling, contrary to our results.

### Experiments

**Figure 2:**
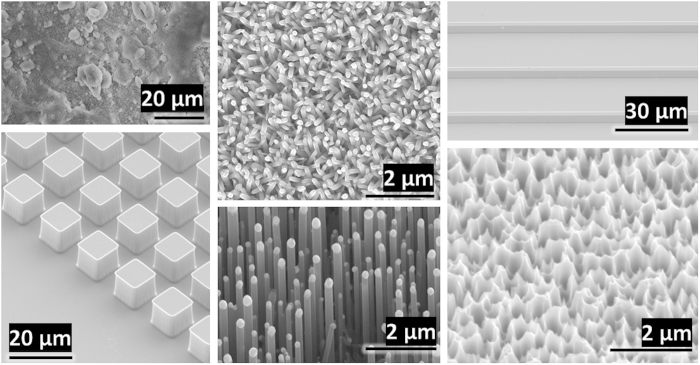
SEM images of the material samples used in our experiments. **Left column:** (top) Polymer/HFS (NC1) composite coating on aluminum substrate, (bottom) silicon square microposts. **Middle column:** (top) zinc oxide nanorods on silicon substrate, (bottom) silicon nanowire forest. **Right column:** (top) silicon microgrooves, (bottom) silicon nanograss.

Physical experiments are conducted to establish the viability of keeping immersed surfaces dry. Different types of samples used include polymer/HFS (NC1), polymer/PTFE (NC2), zinc oxide nanorods, silicon nanograss, silicon microposts, silicon microgrooves, and silicon nanowire forests. Refer to Supplementary Section 3 for fabrication details of each sample. Each sample has some protruding “structure” (e.g. pillars, particles, etc.). The spacing between structures, structure width, structure height, and material contact angle for these samples are reported in Table 1. Scanning electron microscope (SEM) images of the samples before immersion are shown in Fig. 2.

Aging, degassing, and imaging experiments are used to verify the role of nanoscale roughness on maintaining dry immersed surfaces. Results for each experiment are reported in Table 2 and summarized below. Experimental details are provided in Supplementary Sections 4-5, Supplementary Table S1, and Supplementary Figures S7-S12.

### Aging experiments

**Figure 3:**
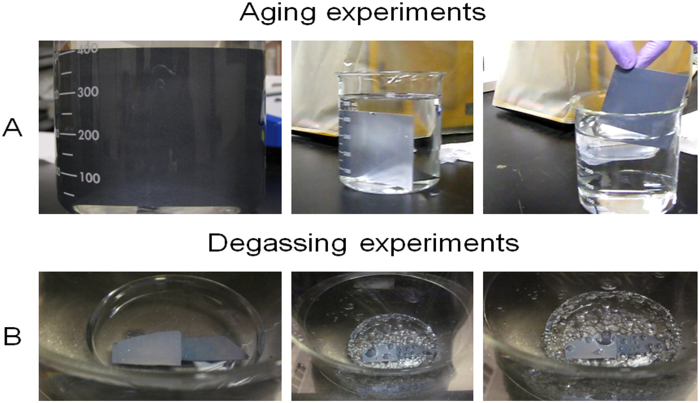
Experiments used to validate non-wetting behavior under water. (**A**) Anodized aluminum substrate coated with PVDF/PMMA and silica nanoparticles (polymer/HFS (NC1)) after 127 days under water. The left image was taken orthogonally to the sample surface; the middle image is a side view that reveals a sheen caused by the thin gas layer between the surface and the water; the right image shows a dry sample upon retrieval from the bath. (**B**) Process of degassing air from the zinc oxide nanorods sample.

Samples are immersed in a beaker of deionized water and shielded from external debris by covering the beaker top. Small holes are made in the cover to keep the system open to the environment. The optical property of total internal reflection is used to distinguish a state where there is a significant gas phase between the liquid and the solid surface. Samples are then removed from the beaker and tested for hydrophobic retention via water droplets. Surfaces that remained practically dry under water are defined as those that did not retain any water film when removed from the water. Figure. 3A shows the polymer/HFS (NC1) coating consisting of PVDF/PMMA (polymer matrix) and silica nanoparticles (filler) on an anodized aluminum substrate.

### Degassing experiments

Samples are immersed in a beaker of water, and then placed into a vacuum desiccator to remove dissolved air. Samples are additionally degassed in a vacuum oven using a similar procedure. If surfaces maintain total reflection sheen *and* come out dry, we conclude that wetting was prevented even after air was depleted from the roughness valleys. This implies that the liquid does not condense in the roughness valleys; instead, the valleys remain dry with presumably the vapor phase in it. The degassing process for the zinc oxide nanorods sample is shown in Fig. 3B. During the degassing process, it is clear when pockets of air are being removed from the surface. These pockets are visible to the naked eye, and coalesce into larger pockets of air. This continued until the pockets were released from the surface into the ambient liquid. We determined the water to be degassed when the air pockets stopped forming near the surface.

The degassing experiments are implemented for shorter times than the aging experiments simply because they required power to run. The vacuum desiccator experiments use a medium-sized chamber that we are able to run for several days. The vacuum oven requires a significant amount of resources to run, and we are not able to leave the vacuum oven running overnight. In Table 2 the reported observations reflect the sample’s final state at the conclusion of the experiment. If a sample appears to become wet with time, the experiment is continued until the sample has fully wetted. For the zinc oxide nanorods samples in the vacuum desiccator, there was no indication (from an undiminished surface sheen) the observed dry state was going to change, hence the experiment was terminated after three days. Some samples, such as the silicon microposts in the vacuum desiccator experiment or the polymer/PTFE (NC2) coating in the vacuum oven experiment, are subject to two rounds of testing. For the first test, the silicon microposts samples with 5 μm and 25 μm spacing appear dry after five days. A second test showed the surfaces became wet within three days. The polymer/PTFE (NC2) coated sample demonstrated similar behavior, remaining dry (four hours) and subsequently becoming wet after five days of immersion.

### Imaging the water-solid interface

**Figure 4:**
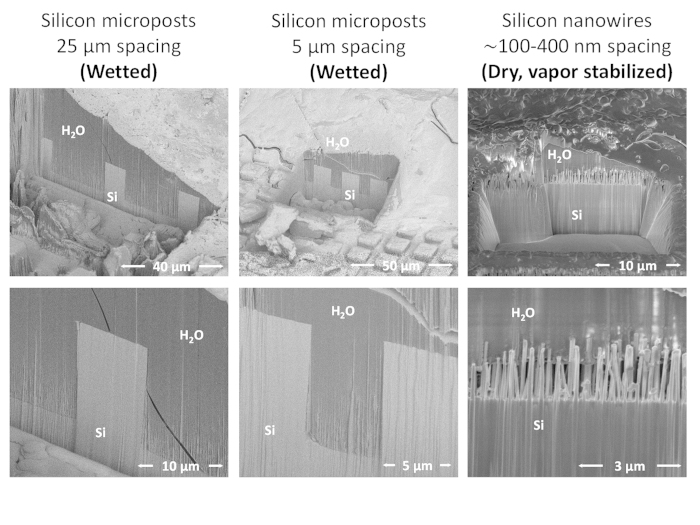
Direct nanoscale imaging of water-solid interfaces. **Left:** Wetted surface with 25 μm pillar spacing. **Middle:** Wetted surface with 5 μm pillar spacing. **Right:** Dry surface with sub-micron pillar spacing. Abbreviations: Frozen water (H_2_O), Silicon substrate (Si).

Direct cross-sectional imaging of water-solid interfaces using cryostabilization, in combination with, cryogenic Focus Ion Beam milling and SEM imaging was recently demonstrated for liquid droplets^44^. In the present work, we adapted the same technique to image water-solid interfaces of superhydrophobic surfaces submerged below a few millimeters of degassed water. Images of the frozen water-solid substrate interface and its dependence on surface roughness spacing is shown in Fig. 4. Liquid invasion is observed for micron-scale roughness spacing, whereas, no invasion is observed for nanometer scale roughness spacing, as predicted.

## Discussion

In each experiment, immersed surfaces with hundreds of nanometer or less spacing remained practically dry. Samples with micron-size feature spacing became wet. The only discrepancy comes from the polymer/HFS (NC1) and polymer/PTFE (NC2) samples. We attribute this to the hierarchical structure of the coatings. The coatings consist of nanoscale spacing on the order of the particle size^45^ (10 nm for HFS, 260 nm for PTFE), where the particles cluster together. On the microscale, spacing of tens of microns can be observed using surface profilometry (see Supplementary Figure S12). Despite being wet, the polymer/PTFE (NC2) sample maintained a silver sheen when immersed. From this, we infer the nanoscale structure is dry in both polymer/PTFE (NC2; 260 nm particles) and polymer/HFS (NC1; 10 nm particles) samples. However, the larger scale structures in these two samples may have been wetted to varying degrees due to different ranges of the length scales involved.

In addition to our experiments, others have observed consistent results in air-retaining insect surfaces. Balmert *et al.*^15^ conducted immersion experiments with air-retaining insect surfaces. Surface roughness on these insects is a result of hair spacing. Insect surfaces that remained dry the longest all had hair spacing of hundreds of nanometers or less, as predicted here.

## Conclusion

Observations of air-retaining insect surfaces, experiments with fabricated surfaces, and molecular dynamics simulations have all shown support for our proposition that sub-micron or smaller scale roughness is essential to maintaining dry surfaces under water. Small length scale roughness is necessary for stabilizing the vapor phase of water, and may serve as precedence for achieving general phase control of fluids using rough surfaces.

## Methods

### Molecular dynamics simulation

The simulation consists of 301,228 atoms, 256,857 of which are water. The pore (Fig. 1A) consists of two flat parallel graphene sheets and a carbon nanotube. The Extended Simple Point Charge (SPC/E^43^) water model is used with SETTLE^46^ for rigid bonds. The Lennard Jones (LJ) carbon-carbon interactions are ε_CC_ = −0.0565 kcal mol^−1^ and σ_CC_ = 3.23895 Å. The piston LJ interactions are ε_piston _= −0.1291 kcal mol^−1^ and σ_piston_ = 3.23895 Å. Carbon hydrophobicity is tuned using the oxygen-carbon LJ well-depth^47^, i.e. ε_OC _= −0.0599 kcal mol^−1^ for hydrophobic surfaces and ε_OC_ = −0.1205 kcal mol^−1^ for hydrophilic surfaces. Note: LJ well-depths in NAMD are negative by convention. Remaining non-bonded cross-interactions are defined by the Lorentz-Berthelot mixing rules. A cutoff radius of 12.0 Å and switch distance of 10.0 Å is used for all non-bonded interactions. The Particle Mesh Ewald algorithm calculated full electrostatic interactions every time step. A constant temperature is maintained using a Langevin thermostat^48^ with a damping coefficient of 0.01 ps^−1^. Carbon surface atoms are fixed, and piston atoms are constrained with a harmonic spring in the *x-y* plane using a force constant of 10 kcal mol^−1^. Water within the nanopore is thermally equilibrated for at least 5 ns, with no applied pressure. For the half-filled nanopore simulations, atom velocities are reassigned during the initial configuration. This is done to prevent full wetting due to inertia from a prior state. The contact angle is measured in accordance with Ref. 49 using bin sizes of 3.5533 Å fitted with a third order polynomial over 462 frames (924 picoseconds). See Supplementary Section 2 for details.

### Material fabrication

Fabrication procedures for each material sample can be found in Supplementary Section 3.

### Degassing experiments

The vacuum desiccator (420220000 Space Saver Vacuum Desiccator 190 mm Clear) reached a target pressure of 21.33–26.34 kPa during the day. The vacuum pump ran intermittently for 5–10 minutes, and then turned off for three hours. This occurred throughout the workday. At night, the pump is turned off while the vacuum desiccator remained closed. The chamber pressure increased overnight to 47.37 kPa the next morning due to leakage. Samples are additionally degassed in a vacuum oven (Model 281A Isotemp Vacuum Oven by Fisher Scientific) using a similar procedure as the vacuum desiccator. The pressure of the vacuum oven is kept at 2.0 kPa, which is below the boiling point of water. Samples are left in the closed oven over night and further degassed the following day.

## Additional Information

**How to cite this article**: Jones, P. R. *et al.* Sustaining dry surfaces under water. *Sci. Rep.* **5**, 12311; doi: 10.1038/srep12311 (2015).

## Supplementary Material

Supplementary Information

## Figures and Tables

**Table 1:** Material properties of surfaces used in the experiments.

**Material**	**Spacing between structures**	**Structure width**	**Structure height**	**Material contact angle**§ 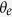
Polymer/HFS (NC1)	O(10 nm) - O(10 μm)†	10 nm‡	Hierarchical†	N/A*
Polymer/PTFE (NC2)	O(260 nm) - O(10 μm)†	260 nm‡	Hierarchical†	N/A*
Zinc oxide nanorods	90–410 nm	40–80 nm	1 μm	110°
Zinc oxide nanorods	20–480 nm	100–150 nm	2 μm	110°
Silicon nanograss	<300 nm^50^	18 nm^50^,^51^	100 nm	110°
Silicon microposts	5 μm	10 μm	10 μm	110°
Silicon microposts	25 μm	10 μm	10 μm	110°
Silicon microgrooves	3 μm	3 μm	5 μm	110°
Silicon microgrooves	12 μm	3 μm	5 μm	110°
Silicon nanowire forest	100–400 nm	50–200 nm	2.5 μm	104°^52^

Material samples consisted of either particle/polymer coatings or pillared-type micro/nano structures.

^§^Contact angle observed on a flat surface (effect due to chemistry, not surface texture).

^†^Hierarchical structure consisting of both nanoscale and microscale surface roughness. The former is of the order of the nanoparticle size, while the latter is of the order of large clusters formed by these particles (as verified by surface profilometry). The nanoscale texture due to the nanoparticles is superimposed on the microscale texture of the coated dry material.

^‡^Nominal diameter of single nanoparticles sprayed onto the surface. These particles may coalesce into larger structures.

^*^The polymer/nanoparticle coatings consist of a composite of different materials, at least one of which is in particle phase. Thus, no smooth surface can be fabricated of the same constituents, making measurement of *θ*_*e*_ not possible.

**Table 2:** Experiment results of immersed surfaces.

**Material**	**Spacing between structures**	**Liquid pressure**	**Observation: Dry/Wet (duration of experiment)**
Aging Experiments			
Polymer/HFS (NC1)^53^	O(10 nm) - O(10 μm)	Ambient	Dry (127 days)
Polymer/HFS (NC1)^53^	O(10 nm) - O(10 μm)	Ambient	Dry (50 days)
Polymer/PTFE (NC2)^53^	O(260 nm) - O(10 μm)	Ambient	Wet (3 days)
Degassing in Vacuum Desiccator			
Polymer/PTFE (NC2)^53^	O(260 nm) - O(10 μm)	All samples: 21.33–26.34 kPa in the daytime, and 47.37 kPa in the nighttime	Wet (30 hours)
Zinc oxide nanorods	90–410 nm		Dry (3 days)
Zinc oxide nanorods	20–480 nm		Dry (3 days)
Silicon nanograss^51^,^54^,^55^	<300 nm		Dry (5 days)
Silicon microposts^54^	5 μm		Wet (3 days)
Silicon microposts^54^	25 μm		Wet (3 days)
Silicon microgrooves	3 μm		Wet (3 days)
Silicon microgrooves	12 μm		Wet (3 days)
Degassing in Vacuum Oven			
Polymer/HFS (NC1)^53^	O(10 nm) - O(10 μm)	2.0 kPa	Wet (5 days)
Zinc oxide nanorods	90–410 nm	2.0 kPa	Dry (1.5 hours)
Zinc oxide nanorods	20–480 nm	2.0 kPa	Dry (1.5 hours)
Silicon nanograss^51^,^54^,^55^	<300 nm	2.0 kPa	Dry (1.5 hours)
Silicon microposts^54^	5 μm	2.0 kPa	Wet (1.5 hours)
Silicon microposts^54^	25 μm	2.0 kPa	Wet (1.5 hours)
Silicon microgrooves	3 μm	2.0 kPa	Wet (1.5 hours)
Silicon microgrooves	12 μm	2.0 kPa	Wet (1.5 hours)
Imaging the water-solid interface			
Silicon nanowire forest^44^,^56^	100–400 nm	A few Torr	Dry (1 minute of degassing)
Silicon microposts^54^	5 μm	A few Torr	Wet (1 minute of degassing)
Silicon microposts^54^	25 μm	A few Torr	Wet (1 minute of degassing)

Aging, degassing, and imaging experiments were conducted on various samples of rough hydrophobic solids immersed in water. Observations for each sample reflect the state of the surface at the conclusion of the experiment, even if non-wetting behavior is initially exhibited. Surfaces with sub-micron or less spacing tended to remain dry, whereas, surfaces with micron spacing became wet, as predicted.
