# Supplementary material for: Sustaining dry surfaces under water
Source: Sci Rep. 2015 Aug 18;5:12311. doi: 10.1038/srep12311 (PMC4539549; doi:10.1038/srep12311)
Supplement: Supplementary Information [file srep12311-s1.doc]

Supplementary Information

Sustaining dry surfaces under water

Paul R. Jones,^1^ Xiuqing Hao,^1^ Eduardo R. Cruz-Chu,^2^ Konrad Rykaczewski,^3^ Krishanu Nandy,^1^ Thomas M. Schutzius,^4^ Kripa K. Varanasi,^5^ Constantine M. Megaridis,^4^ Jens H. Walther,^2,6^ Petros Koumoutsakos,^2^ Horacio D. Espinosa,^1^ Neelesh A. Patankar^1,*^

^1^*Department of Mechanical Engineering, Northwestern University, Evanston, IL, USA.*

^2^*Institute of Computational Science, ETH Zürich, Zürich, Switzerland.*

^3^*School for Engineering of Matter, Transport and Energy, Arizona State University, Tempe, AZ, USA.*

^4^*Department of Mechanical and Industrial Engineering, University of Illinois at Chicago, Chicago, IL, USA.*

^5^*Department of Mechanical Engineering, MIT, Cambridge, MA, USA.*

^6^*Department of Mechanical Engineering, Tech. University of Denmark, Kgs. Lyngby, Denmark*

^*^*E-mail*: n-patankar@northwestern.edu

Contents

[List of supplementary tables and legends 3](#__RefHeading___Toc422210443)

[List of supplementary figures and labels 3](#__RefHeading___Toc422210444)

[1 Heterogeneous nucleation theory 4](#__RefHeading___Toc422210445)

[2 Measuring contact angles in molecular dynamics simulations 7](#__RefHeading___Toc422210446)

[3 Material Fabrication 8](#__RefHeading___Toc422210447)

[3.1 Polymer-nanoparticle composite coatings on aluminum 8](#__RefHeading___Toc422210448)

[3.2 Zinc oxide coated samples 9](#__RefHeading___Toc422210449)

[3.3 Silicon nanowire forest 10](#__RefHeading___Toc422210450)

[3.4 Silicon square microposts array 10](#__RefHeading___Toc422210451)

[3.5 Silicon microgrooves 10](#__RefHeading___Toc422210452)

[3.6 Silicon nanograss 11](#__RefHeading___Toc422210453)

[4 Experimental Methods 11](#__RefHeading___Toc422210454)

[4.1 Aging experiments 11](#__RefHeading___Toc422210455)

[4.2 Repeated aging experiment of the polymer/HFS (NC1) 11](#__RefHeading___Toc422210456)

[4.3 Degassing experiments 12](#__RefHeading___Toc422210457)

[5 Direct nanoscale imaging of water-solid interfaces 12](#__RefHeading___Toc422210458)

[6 Supplementary tables and legends 14](#__RefHeading___Toc422210459)

[7 Supplementary figures and legends 15](#__RefHeading___Toc422210460)

[References 24](#__RefHeading___Toc422210461)

# List of supplementary tables and legends

[Supplementary Table S1: Composition of dispersions used to create coatings 14](#__RefHeading___Toc422210462)

# List of supplementary figures and labels

[*Supplementary Figure S1: Illustration of practically dry surfaces under water.* 15](#__RefHeading___Toc422210463)

[*Supplementary Figure S2: Typical liquid-vapor phase diagram for water.* 16](#__RefHeading___Toc422210464)

[*Supplementary Figure S3: Heterogeneous nucleation theory for water at 300 K.^1,2,5^* 17](#__RefHeading___Toc422210465)

[*Supplementary Figure S4: MD simulations of a hydrophobic pore that is initially unfilled.* 18](#__RefHeading___Toc422210466)

[*Supplementary Figure S5: MD simulations of a hydrophobic pore that is initially half-filled.* 19](#__RefHeading___Toc422210467)

[*Supplementary Figure S6: MD simulations of a hydrophilic pore that is initially unfilled.* 20](#__RefHeading___Toc422210468)

[*Supplementary Figure S7: Aging experiment for immersed polymer/nanoparticle coatings.* 20](#__RefHeading___Toc422210469)

[*Supplementary Figure S8: Zinc oxide coated sample in vacuum desiccator.* 21](#__RefHeading___Toc422210470)

[*Supplementary Figure S9: Immersed silicon microposts/microgrooves samples.* 21](#__RefHeading___Toc422210471)

[*Supplementary Figure S10: Silicon microposts/microgrooves samples in vacuum desiccator.* 22](#__RefHeading___Toc422210472)

[*Supplementary Figure S11: Process for direct imaging of the liquid-solid interface.* 22](#__RefHeading___Toc422210473)

[Supplementary Figure S12: Surface profilometry of the polymer/HFS (NC1) coating. 23](#__RefHeading___Toc422210474)

# Heterogeneous nucleation theory

We use heterogeneous nucleation theory for a droplet of liquid condensate. The free energy for nucleation is described by:

ΔG = -V_l_(Δp) + A_lv_σ_lv_ + A_ls_(σ_ls_ – σ_vs_), (S1)

where V_l_ is the liquid condensate volume, A_lv_ is the liquid-vapor surface area, and A_ls_ is the liquid-solid surface area. The surface energies are σ_ls_, σ_vs_, and σ_lv_ for the liquid-solid (ls), vapor-solid (vs), and liquid-vapor (lv) interfaces. We may use the Young equation (ignoring line tension) to obtain σ_ls_ – σ_vs_ = -σ_lv_cosθ_e_, where θ_e_ is the material contact angle. The pressure difference between the liquid condensate (p_l_) and ambient vapor (p_v_) is Δp = p_l_ - p_v_. The Young-Laplace equation for mechanical equilibrium at the liquid-vapor interface requires

p_l_ - p_v_ = 2σ_lv_/r, (S2)

where r is the radius of the condensed droplet. Chemical equilibrium requires

p_v_ = p_sat_ exp[ (ѵ_l_/(RT) ) (p_l_ - p_sat_)], (S3)

where p_sat_ is the saturation pressure, T is the temperature, ѵ_l_ is the specific volume of liquid water, and R is the specific gas constant of vapor. For a droplet of condensate on a flat surface,

V_l_ = (1/3)πr^3^(2+cosθ)(1-cosθ)^2^, (S4)

A_lv_ = 2πr^2^(1-cosθ), (S5)

A_ls_ = πr^2^sin^2^θ, (S6)

where θ is the apparent liquid contact angle.

The normalized free energy for a condensate on a flat surface with θ = θ_e_ = 110° is plotted in Supplementary Figure S3A. The free energy is normalized by ΔG_norm_ = (ΔG-ΔG_min_)/(ΔG_max_-ΔG_min_). Here, ΔG_min_ and ΔG_max_ correspond to the minimum and maximum energy states on the free energy landscape. The parameters and variables in these calculations are chosen to be consistent with those presented in the Manuscript for water at 300 K. They are, p_l_ = 101.325 kPa, p_sat_ = 3.537 kPa[^5^](#_ENREF_5), p_v_ = 3.539 kPa (this follows from equation S3 with R = 461.52 J/kg-K and ѵ_l_ = 1.0035×10^-3^ m^3^/kg), and σ_lv_ = 71.7 mN/m[^5^](#_ENREF_5). The energy barrier for nucleation (Supplementary Figure S3A) occurs when the free energy is maximized. The condensate radius associated with this barrier is the critical radius r_e_ = 2σ_lv_/Δp = 1.47 µm.

For vapor-stabilization to occur inside the pore, we need to prevent nucleation within the pore. As observed in Supplementary Figure S3A, nucleation would occur when the condensate size is such that r = r_e_ = 2σ_lv_/Δp. If we decrease the radius of the pore r_p_ to be less than r = r_e_, then a condensate with critical radius r_e_ will start wetting the vertical walls of the pore. This is illustrated in Supplementary Figure S3B. For the cylindrically pored system and the condensate configuration shown in Supplementary Figure S3B (II), we have

ΔG = -V_l_(Δp) + A_lv_σ_lv_ - A_ls_σ_lv_cosθ_e_, (S7)

V_l_ = (1/3)πr^3^(2+cos(θ - 90°))(1-cos(θ - 90°))^2^ + πr_p_^2^h, (S8)

A_lv_ = 2πr^2^(1-cos(θ - 90°)), (S9)

A_ls_ = 2πr_p_h + πr_p_^2^, (S10)

where h is the vertical height of the liquid-vapor-solid triple line from the bottom of the pore. For simplicity, we consider only equations that represent the free energy change of the confined droplet, and do not include the formation of the meniscus of the pooled liquid at the top of the pore. From geometry we can also write[^4^](#_ENREF_4)

D = 2r_p_ = -2rcosθ, (S11)

where D is the diameter of the pore. Using equation (S11), we can vary the diameter of the pore to change the apparent contact angle θ of the condensate with respect to the vertical walls of the pore (Supplementary Figure S3B). The free energy landscape for nucleation can then be plotted as a function of the condensate size r and the apparent contact angle θ. The energy landscape is shown in Supplementary Figure S3C.

It is seen from Supplementary Figure S3C that the energy barrier for nucleation occurs when the pore diameter D = -2r_e_cosθ_e_ = 1.0 µm, i.e. when θ = θ_e_ = 110° and the droplet size r = r_e_ = 2σ_lv_/Δp = 1.47 µm. The energy landscape shows, for example, that when r = r_e_ and θ > θ_e_ the free energy will decrease, and the condensate would continue to grow, whereas when r = r_e_ and θ < θ_e_ the free energy will decrease, as the condensate would shrink. For a condensate with r = r_e_ = 2σ_lv_/ Δp, the condition θ < θ_e_ to prevent condensation occurs when D < -2r_e_cosθ_e_ according to equation (S11). This criterion to prevent condensation inside the pore is thus identical to the impalement criterion in equation (1) of the Manuscript (note r_e_ = 2σ_lv_/Δp).

In summary, with r = r_e_ and θ > θ_e_ > 90°, we get p_l_ > p_v_ – 4σ_lv_cosθ_e_/D and the surface will wet, whereas with r = r_e_ and θ_e_ > θ > 90°, we get p_l_ < p_v_ – 4σ_lv_cosθ_e_/D and the surface will remain dry. Thus, when designing surfaces to remain dry under water, we can simultaneously prevent condensation from occurring within the pore, as well as liquid invasion by using equation (1). Finally, we remark that the criterion to prevent condensation in the pore is derived based on a configuration depicted in Supplementary Figure S3B. An analysis, accounting for all possible condensation pathways has not been done. This is no different from the transition criterion for impalement of liquid into roughness grooves. There too, the impalement criterion has been derived based on a simplified model geometry.[^6^](#_ENREF_6)

# Measuring contact angles in molecular dynamics simulations

A single contact angle, obtained from the vapor-stabilization case at 300 K, 107.79 bar pressure is used to estimate the critical contact angle used in equation (1). Oxygen atoms at the liquid-vapor interface (2 Angstrom thick) were superimposed onto a single plot over the last 924 picoseconds of simulation. The interfacial oxygen were discretized in accordance with Ref[^7^](#_ENREF_7) using bin sizes of 3.5533 Å. The contact angle was calculated by fitting a third order polynomial to the average radius within each bin. Using this method, the local contact angle was 119.4°.

To estimate the error associated with this measurement we rearrange equation (1) in terms of σ_lg_. The surface energy, σ_lg_ = -(p_l_-p_g_)D(4cos θ)^-1^ = -(Δp)D(4cos θ)^-1^. At the same temperature we expect σ_lg_ to remain constant, thus, σ_lg_ = -(Δp)_Out_D(4cos θ_Out_)^-1^ = -(Δp)_In_D(4cos θ_In_)^-1^ where (Δp)_Out_ = p_l_-p_g_ and p_l_ is the pressure for vapor-stabilization. (Δp)_In_ = p_l_-p_g_ where p_l_ is the pressure for liquid invasion. The contact angles, θ_Out_ and θ_In_ correspond with the vapor-stabilization pressure and liquid invasion pressure respectively. Approximating the contact angle at the point of liquid invasion gives θ_In_ = cos^-1^[((Δp)_In_ /(Δp)_Out_)cos θ_Out_]. Using vapor-stabilization and liquid invasion pressures at each isotherm yields θ_In_ = 125.51°, 126.92°, 128.51°, and 124.18°. The maximum difference between the calculated contact angle of 119.44° and the estimated contact angles for invasion is 9.07°. For this report, we estimate θ_e_ as 119.44° (accurate to within 9.07°).

# Material Fabrication

Polymer/nanoparticle coated samples were fabricated by the University of Illinois at Chicago (UIC). Zinc oxide coated silicon samples were fabricated by Xi’an Jiaotong University. Silicon nanowire forest, silicon square microposts array, and silicon microgrooves samples were fabricated by the Massachusetts Institute of Technology (MIT) and the National Institute of Standards and Technology (NIST).

## Polymer-nanoparticle composite coatings on aluminum

Materials: Acetone (ACS reagent, ≥ 99.5%), *N*-methyl-2-pyrrolidone (NMP, 99.5 wt. %), silicon dioxide nanopowder (99.5% metals basis, 5-15 nm), poly (methyl methacrylate) powder (PMMA, ~996,000 Da), and poly (tetrafluoroethylene) powder (PTFE, < 1 μm) were all obtained from Sigma-Aldrich. Poly (vinylidene fluoride) powder (PVDF, melt viscosity: 2,350-2,950 Pa s, melting point: 155-160 ºC) was obtained from Alfa-Aesar. Hydrophobic fumed silica (HFS, specific surface area (BET): 170±20 m^2^ g^-1^; Aerosil® R 9200) was obtained from Evonik Industries. Previous analysis showed the primary feature size of PTFE particles to be 260±54 nm[^8^](#_ENREF_8).

Fabrication: Separately, 10 wt. % stock solutions of PVDF and PMMA were generated by dissolving PVDF and PMMA powder in NMP and acetone, respectively, under slow mechanical mixing at room temperature overnight. In a typical case, a PTFE or HFS suspension was formed by combining 0.75 g of particles and 11 g of acetone in a 20 mL vial; this entire suspension was then bath sonicated (Cole-Parmer, 70 W, 42 kHz) for several minutes. Once a stable suspension was formed, 0.62 g of 10 wt. % PVDF and 0.62 g of 10 wt. % PMMA were added to it, and the entire dispersion was shaken mechanically at room temperature. The dispersion was then spray deposited with an airbrush (Paasche VL siphon feed, 0.73 mm spray nozzle) onto aluminum plates (i.e., mirror finished anodized aluminum) using compressed air (-2.8 bar) at a fixed distance of ~10 cm. Initially the coatings were dried with a heat gun (Proheat® Varitemp® PH-1200, 1300 W max) for a minute and were then placed in an oven at 150 ºC for 60 min to completely dry, thus forming a superhydrophobic, self-cleaning coating. See Supplementary Table S1 for a description of the individual ingredients and their concentration in the dispersions used for spray. The previously mentioned procedure refers to coatings NC1 (HFS containing) and NC2 (PTFE containing).

## Zinc oxide coated samples

Hydrothermal growth: First, a zinc oxide (ZnO) seed crystal layer of about 30~50 nm is prepared on the silicon surface by radio frequency (RF) magnetron sputtering system (explorer 14, Denton Vacuum, USA). The parameters of the sputter are as follows: power 150 W, flowrate Ar 20 sccm, and sputtering time 6 min. Second, ZnO nanorod arrays are synthesized on a seed crystal layer by hydrothermal method. The aqueous solution is a mixture of zinc nitrate hexahydrate (Zn(NO_3_)_2_·6H_2_O, 25 mM or 50 mM), hexamethylenetetramine (HMT, C_6_H_12_N_4_, Zn^2+^ and HMT are kept at the same 1:1 molar ratio), and deionized water (100 mL). ZnO nanorods were then synthesized at a temperature of 95℃ for 1-3 hours.

Surface treatment: A 1% solution of F8261 (1H,1H,2H,2H-perfluoroalkyltriethoxysilanes) and methanol were used to chemically treat the surface. The surface was immersed in the solution for 26 hours, and subsequently heated for 1.5 hours at a temperature of 150 ℃. Roll-off tests show the two samples are superhydrophobic, as indicated by water droplets easily rolling off the sample surface.

## Silicon nanowire forest

Silicon nanowires were grown in a custom-designed horizontal hot-walled chemical vapor deposition reactor at 850°C using a SiCl_4_/H_2_/N_2_ gaseous mixture. Gold nanoparticles formed on a Si(111) substrate by annealing a 5 nm thick gold film were used to catalyze the vapor-liquid-solid growth. To produce the superhydrophobic surface, grown nanowires with height of about 2.5 µm and diameters between 50 nm and 200 nm were modified using vacuum deposition of 1H,1H,2H,2H-Perfluorodecyltrichlorosilane (Alfa Aesar). Further details of the nanowire growth and surface modification can be found elsewhere.

## Silicon square microposts array

350 μm thick Si n-(100) substrates were patterned via photolithography and etched via Deep Reactive Ion Etching to obtain arrays of 10 μm tall square microposts with widths of 10 μm and edge-to-edge spacing of 5 μm and 25 μm. After cleaning the samples with Piranha solution, the samples were coated with octadecyltrichlorosilane (Sigma Aldrich) using a solution deposition method, rendering the surface hydrophobic.[^11^](#_ENREF_11)

## Silicon microgrooves

Two silicon microgroove samples were fabricated using the same procedure as the silicon square micropost array. The groove thickeness, height, and spacing was 3 μm, 5 μm, 3 μm for the first sample and 3 μm, 5 μm, 12 μm for the second sample.

## Silicon nanograss

Silicon nanograss samples were fabricated using plasma, consisting of O_2_ and SF_6_. This produced surface texture with estimated roughness spacing of 100 nm, and 200 nm heights. The samples were coated with octadecyltrichlorosilane (Sigma Aldrich) using a solution deposition method. Details of the fabrication procedure can be found elsewhere.[^11-13^](#_ENREF_11)

# Experimental Methods

## Aging experiments

The two polymer/nanoparticle coated surfaces appeared matte black, when viewed from the normal direction, at all times in the experiment. When initially submerged, both substrates appeared to have a trapped layer of gas over their surface. The layer of gas in the Polymer/PTFE (NC2) sample appeared significantly reduced within a few hours. After seventy-two hours, no presence of the trapped gas could be observed. In comparison, the polymer/HFS (NC1) sample showed almost no visual change in the quality of the trapped gas layer over a comparable period. The polymer/HFS (NC1) sample maintained the gas layer for 127 days (termination of experiment). The sample was then removed from the beaker and visually inspected. The majority of the sample remained dry, despite having been immersed in water for over four months. This was demonstrated using a pipette to place drops of water on the surface held at an angle; the droplets rolled off the surface upon contact

## Repeated aging experiment of the polymer/HFS (NC1)

The aging experiment with polymer/HFS (NC1) was repeated for 50 days using a different sample with the same structure spacing provided by UIC. The results were consistent with the previous samples subjected to water exposure for 127 days.

## Degassing experiments

The polymer/PTFE (NC2) sample was subjected to five rounds of degassing in the vacuum desiccator. The polymer/HFS (NC1) coated sample was degassed twice over a period of four hours in the vacuum oven. The silicon nanograss sample was degassed twice in the vacuum oven.

# Direct nanoscale imaging of water-solid interfaces

See Supplementary Figure S11 for a schematic of the imaging procedure. The direct interface imaging experiments were carried out using an FEI Nova Nanolab 600 Dual Beam equipped with a Quorum PP2000T cryo-transfer system. About 7 mm by 7 mm pieces of the superhydrophobic surfaces were attached using double stick copper tape to a 10 mm diameter copper stub. About 5 mm tall piece of 9.5 mm (3/8 inch) inner diameter vinyl tubing was fitted around the copper stub. This tubing provided a watertight seal around the copper stub, creating a temporary water container that protruded about 3 mm above the sample. This temporary sample holder was submerged in a beaker filled with 30 mL of distilled water, which was placed in the vacuum desiccator used to make liquid nitrogen slush. The pressure within the chamber was reduced using a roughing pump until intensive bubble formation was observed. To prevent the water from freezing, bubbling intensity was reduced by leaking in nitrogen gas. After one minute of degassing, the vacuum desiccator was vented and the sample holder was carefully removed from the water beaker. The 3 mm water layer above the sample was subsequently frozen by placing the bottom part of the copper stub in contact with liquid nitrogen. We note that in our experiments the freezing rate was slower than during liquid nitrogen plunge freezing. Thus, some deformation of the liquid-gas-solid interface is expected due to crystallization.

The copper stub with ice-covered sample was attached to cryo-FIB/SEM shuttle and plunge frozen in liquid nitrogen slush. This proved to be necessary to prevent sticking of the transfer rod to the shuttle due to condensate freezing around the rod’s thread. Next, the frozen sample was moved into the transfer chamber. The chamber was pre-evacuated to a pressure of 10^-3^ Pa and pre-cooled to a temperature of -180°C. To prepare the sample for FIB/SEM imaging, the thick ice layer was mechanically broken up using a metal cryo-manipulator and partially sublimated by raising the sample temperature to -90°C for a period of twenty to thirty minutes. To prevent charging of the sample, in situ 20 nm to 30 nm platinum depositions were achieved by plasma sputtering in Argon gas environment with 8 mA current for two minutes. After transfer to the cryo-FIB/SEM chamber, the samples were exposed to a 10 s pulse of C_9_H_16_Pt gas precursor at 28°C. The resulting hundreds of nanometers to a few micrometers thick coating was cured by exposure to ion beam with energy and current of 30 keV and 2.6 nA, respectively. This additional coating (whitish-grey in color, covering the outermost surface shown in Figure 4 of the main text) aided uniform milling during the cross-sectioning step.

FIB milling was performed with sample tilt of 52°, ion beam energy of 30 keV, an ion current of 0.093 nA to 21 nA, and a dwell time of 1 µs per pixel. The structure cross-sections were obtained by first FIB milling deep trenches at an ion beam current of 21 nA. Subsequently the surfaces were polished at 0.48 nA to 2.6 nA. All cross-sections were imaged at 52° tilt in the objective lens immersion mode using backscattered electrons with electron beam energy and current of 1 keV and 0.46 nA, respectively.

# Supplementary tables and legends

Supplementary Table S1: Composition of dispersions used to create coatings

| **Ingredient** | **NC1**  **Concentration (wt. %)** | **NC2**  **Concentration (wt. %)** |
| --- | --- | --- |
| PVDF | 0.5 | 0.5 |
| PMMA | 0.5 | 0.5 |
| HFS | 5.8 | 0.0 |
| PTFE | 0.0 | 5.8 |
| Acetone | 88.9 | 88.9 |
| NMP | 4.3 | 4.3 |

# Supplementary figures and legends


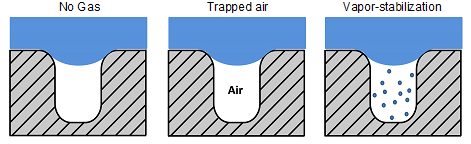


Supplementary Figure S1: Illustration of practically dry surfaces under water.

Left: The roughness groove is not occupied by a gas. Middle: Air is trapped within the roughness groove. Right: Vapor fills the roughness groove and is in equilibrium with the overlying liquid.


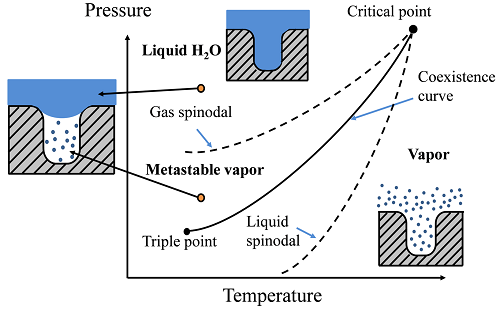


Supplementary Figure S2: Typical liquid-vapor phase diagram for water.

The textured surface is superimposed on this diagram to indicate the phase of water within surface grooves. Here, we are designing for a metastable vapor to exist within surface grooves, and a stable liquid outside the grooves.

| A |  |
| --- | --- |
| B |  |
| C |

Supplementary Figure S3: Heterogeneous nucleation theory for water at 300 K.

**(A)** Nucleation of condensate on a flat surface with θ_e_=110°. The free energy is normalized by ΔG_norm_ = (ΔG-ΔG_min_)/(ΔG_max_-ΔG_min_), where ΔG_min_ is the minimum energy state, and ΔG_max_ is the maximum energy state on the free energy landscape. The energy barrier ΔG_norm_(r_e_) may be exceeded when the condensate’s critical radius reaches r_e_ = 2σ_lv_/Δp = 1.47 µm. Note: ambient vapor is not shown near the condensate. **(B)** Droplet nucleation of critical size (r_e_ = 1.47 µm) within a cylindrical pore of radius r_p_. (I) Condensate does not touch vertical pore walls (r_p_ > r_e_). (II) Condensate touches pore walls (r_p_ < r_e_). (C) Normalized free energy for a condensate within a cylindrical pore. (I) Energy landscape for θ ϵ [95°, 150°], r ϵ [0,4r_e_]. (II) Magnified view of the energy landscape at the saddle point barrier. The pore diameter, or equivalently the apparent liquid contact angle, is changed according to equation (S11). The energy barrier ΔG_norm_(θ_e_, r_e_) occurs when the pore diameter D = 1 µm and consequently θ = θ_e_ = 110°, with critical droplet size r_e_ = 2σ_lv_/Δp = 1.47 µm.


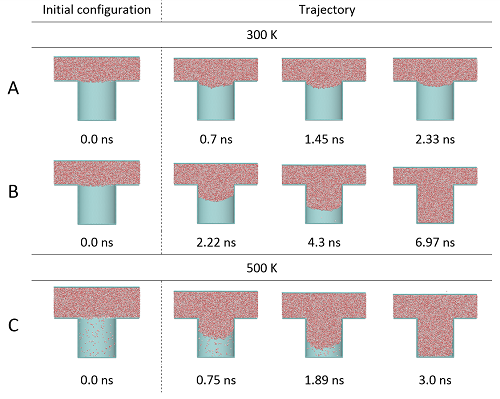


Supplementary Figure S4: MD simulations of a hydrophobic pore that is initially unfilled.

**(A)** Hydrophobic pore demonstrating non-wetting at 300 K, 107.79 bar applied pressure.

**(B)** Hydrophobic pore that fully wets the surface at 300 K, 127.39 bar applied pressure.

**(C)** Hydrophobic pore that fully wets the surface at 500 K, 78.39 bar applied pressure.


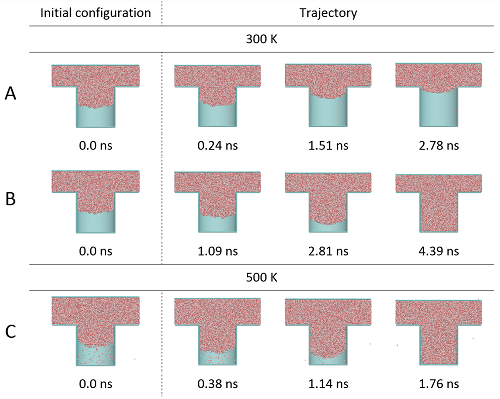


Supplementary Figure S5: MD simulations of a hydrophobic pore that is initially half-filled.

**(A)** Hydrophobic pore demonstrating non-wetting at 300 K, 107.79 bar applied pressure.

**(B)** Hydrophobic pore that fully wets the surface at 300 K, 127.39 bar applied pressure.

**(C)** Hydrophobic pore that fully wets the surface at 500 K, 78.39 bar applied pressure.


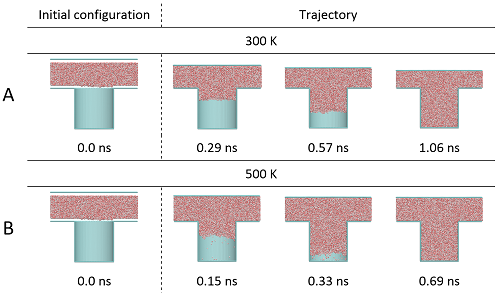


Supplementary Figure S6: MD simulations of a hydrophilic pore that is initially unfilled.

**(A)** Hydrophilic pore that fully wets the surface at 300 K, 0 bar applied pressure.

**(B)** Hydrophilic pore that fully wets the surface at 500 K, 0 bar applied pressure.

|  | **(A) Front view** | **(B) Angled view** |
| --- | --- | --- |
| *Polymer/HFS (NC1)* | 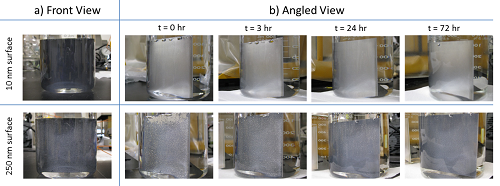 | |
| *Polymer/PTFE (NC2)* |

Supplementary Figure S7: Aging experiment for immersed polymer/nanoparticle coatings.

**(A)** Front view of submerged surfaces. **(B)** Angled view of submerged surfaces at various times after initial immersion.


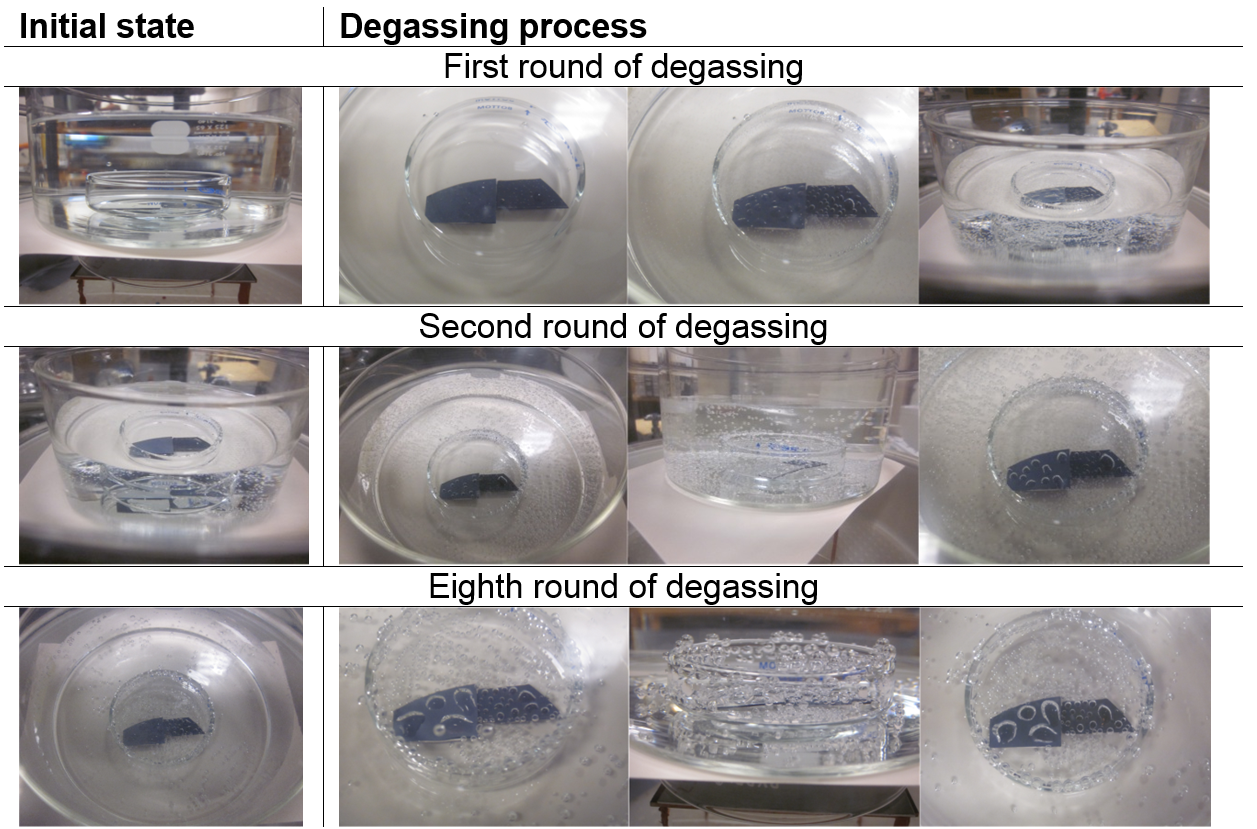


Supplementary Figure S8: Zinc oxide coated sample in vacuum desiccator.


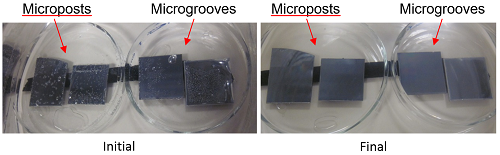


Supplementary Figure S9: Immersed silicon microposts/microgrooves samples.

Samples are shown after three days of degassing in the vacuum desiccator.


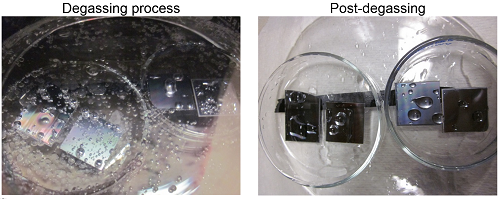


Supplementary Figure S10: Silicon microposts/microgrooves samples in vacuum desiccator.

The samples are wet after degassing in the vacuum oven.


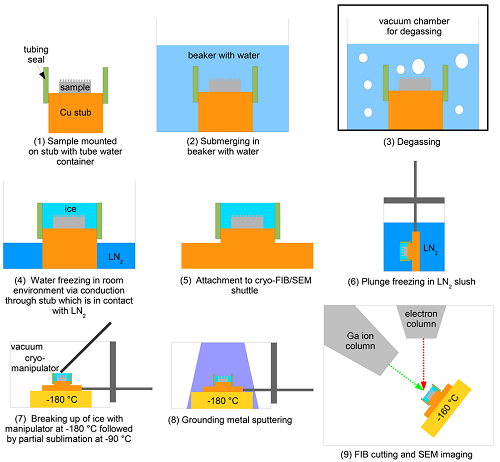


Supplementary Figure S11: Process for direct imaging of the liquid-solid interface.


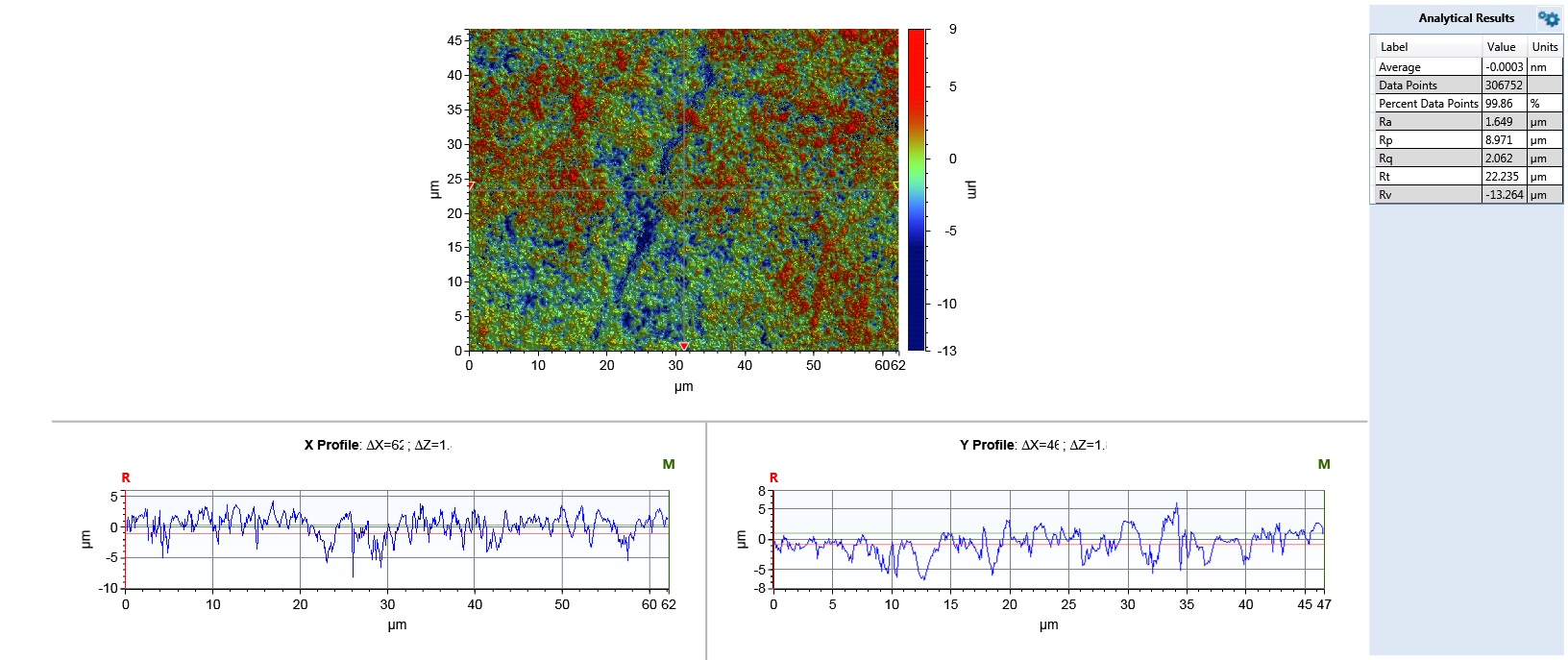


Supplementary Figure S12: Surface profilometry of the polymer/HFS (NC1) coating.

# References
